# Synthetic approach to borrelidin fragments: focus on key intermediates

**DOI:** 10.3762/bjoc.21.91

**Published:** 2025-06-12

**Authors:** Yudhi Dwi Kurniawan, Zetryana Puteri Tachrim, Teni Ernawati, Faris Hermawan, Ima Nurasiyah, Muhammad Alfin Sulmantara

**Affiliations:** 1 Research Center for Pharmaceutical Ingredient and Traditional Medicine, National Research and Innovation Agency (BRIN), Tangerang Selatan, Banten 15314, Indonesia; 2 Department of Chemistry, Faculty of Mathematics and Natural Sciences, Universitas Negeri Semarang, Semarang, Central Java 50229, Indonesia

**Keywords:** borrelidin analogs, borrelidin derivatives, borrelidin fragments, borrelidin synthesis, fragment-based synthesis

## Abstract

Borrelidin, a naturally occurring antibiotic, has attracted considerable interest due to its diverse biological activities and complex molecular architecture. Although extensive research has explored its pharmacological properties and various synthetic approaches, significant challenges remain in the efficient synthesis of borrelidin and its analogs. Existing literature largely focuses on total synthesis, bioactivity, and structural modifications, leaving a notable gap in fragment-focused synthesis, particularly for its intricate substructures. This review seeks to address this gap by offering a detailed examination of borrelidin fragment synthesis, highlighting key challenges and innovative strategies involved. By pinpointing unresolved synthetic hurdles, this work advocates for a fragment-focused approach as a crucial step toward advancing borrelidin research and expanding its potential applications.

## Introduction

**Figure 1:**
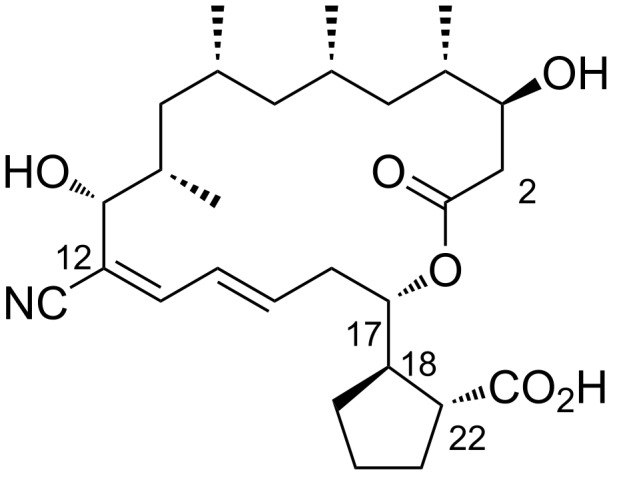
Chemical structure of borrelidin (**1**).

Borrelidin (Figure 1), a distinctive 18-membered ring macrolide, was first isolated from *Streptomyces rochei* by Berger et al. in 1949 [1]. This antibiotic, also known for its anti-Borrelia activity and ability to enhance penicillin’s effects, was structurally elucidated in 1967 by Keller-Schierlein [2]. Since then, borrelidin has been recognized for its potential as a cancer therapeutic [3–5], exhibiting anti-inflammatory [6], anti-angiogenic [7–9], antimicrobial [10], antifungal [11–13], and antimalarial activities [14–15]. Since then, borrelidin has been recognized for its potential as a cancer therapeutic, exhibiting anti-inflammatory, anti-angiogenic, antimicrobial, antifungal, and antimalarial activities. While Keller-Schierlein first determined its chemical structure, Anderson later confirmed its absolute configuration using X-ray crystallography [16].

During the screening of a marine or hypersaline environment natural products library to identify potent drugs, a putatively novel metabolite co-produced with borrelidin was discovered, expanding the potential for new borrelidin derivatives. This led to the formation of the so-called “borrelidin family” (Table 1), with derivatives named alphabetically starting from the original borrelidin **1**, designated as borrelidin A (Table 1, entry 1). A rare nitrile moiety at C12 of the macrolide ring in borrelidin A is present in most members of the borrelidin family (Table 1, entries 4, 5, 7–11, 13–16, and 18), except for borrelidin B, *N*-acetylborrelidin B, borrelidin CR2, borrelidin I, and borrelidin N (Table 1, entries 2, 3, 6, 12, and 17). Borrelidin B (Table 1, entry 2), a tetrahydroborrelidin derivative with an aminomethyl group instead of the nitrile in position 12 of the macrolide, was isolated from the marine-derived *Streptomyces* sp*.* RL09-241-NTF-B strain [17]. The discovery of borrelidin B, along with the novel introduction of an *N*-acetyl group in borrelidin B (*N*-acetylborrelidin B, Table 1, entry 3), expanded the borrelidin family. *N*-Acetylborrelidin B was obtained from *Streptomyces mutabilis* sp. MII [18], a marine strain from the Red Sea (Egypt), further enriching the borrelidin series.

A halophilic actinomycete strain (HYJ128) from Jeung-do Island (Shinan-gun, Jeollanamdo, Korea), belonging to the genus *Nocardiopsis*, inhabits a hypersaline saltern and was found to produce a series of new polyketide-derived macrolides with hydroxy groups at C20 or C7, identified as borrelidins C–E (Table 1, entries 4, 7, and 8) [19]. Borrelidins CR1 and CR2 (Table 1, entries 5 and 6), amide-containing congeners, were also isolated through bioassay-guided fractionation and purification of marine microorganisms from Costa Rica [20]. Borrelidin CR1 (Table 1, entry 5) was also discovered in *Streptomyces olivaceus* SCSIO LO13 from *Onchidium* sp. (South China Sea), alongside other borrelidin derivatives [21]. Borrelidins F–I (Table 1, entries 9–12) were obtained from *Streptomyces rochei* SCSIO ZJ89, a strain from mangrove-derived sediment in Yalongwan, China [22]. The C14–C15 olefin geometry of borrelidins G and H (Table 1, entries 10 and 11) exhibited a Z-configuration, as confirmed by NOESY correlations. Borrelidins J–L (Table 1, entries 13–15) were isolated from an endophytic *Streptomyces* sp. NA06554 from *Aster tataricus* in Aba County, Sichuan Province, China, along with other borrelidin derivatives, including borrelidin E (Table 1, entry 8) and 12-desnitrile-12-carboxyborrelidin (Table 1, entry 19) [23]. The latter compound, found in both endophytic bacteria and as a product of borrelidin biosynthesis, contributed to the understanding of nitrile formation [24].

**Table 1:** Summary of the borrelidin family: structures, sources, and related literature.

Entry	Name	Structure	Source	Ref.

1	borrelidin A	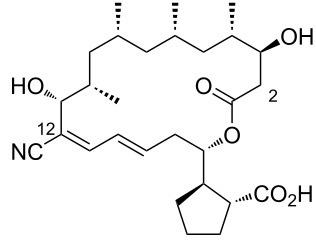 **1**	first encounter was isolated from *Streptomyces* sp. and found to be produced along with other members of the borrelidin family	[1]
2	borrelidin B	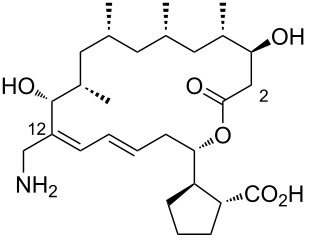 **2**	*Streptomyces* sp. RL09-241-NTF-B	[17]
3	*N*-acetylborrelidin B	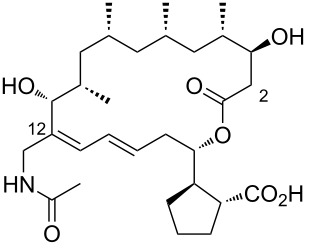 **3**	*Streptomyces mutabilis* sp. MII	[18]
4	borrelidin C	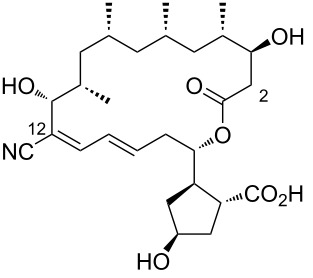 **4**	*Nocardiopsis* sp.	[19]
5	borrelidin CR1	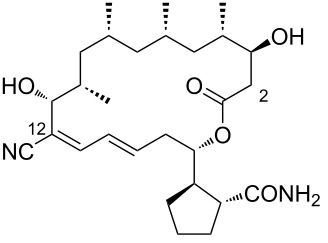 **5**	a terminal unfolded protein response (UPR)-inducing natural extracts (cultivated marine microorganisms) *Onchidium sp.* associated *Streptomyces olivaceus* SCSIO LO13	[20–21]
6	borrelidin CR2	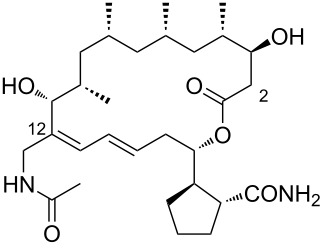 **6**	a terminal unfolded protein response (UPR)-inducing natural extracts (cultivated marine microorganisms)	[20]
7	borrelidin D	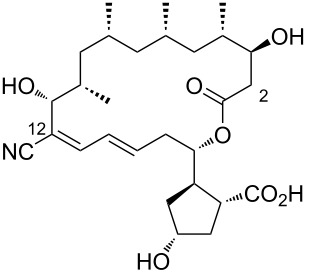 **7**	*Nocardiopsis* sp.	[19]
8	borrelidin E	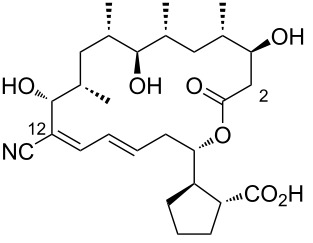 **8**	an endophytic *Streptomyces* sp. NA06554 from *Aster tataricus* marine pulmonated mollusks *Onchidium sp.* associated *Streptomyces olivaceus* SCSIO LO13 *Nocardiopsis* sp.	[19,21,23]
9	borrelidin F	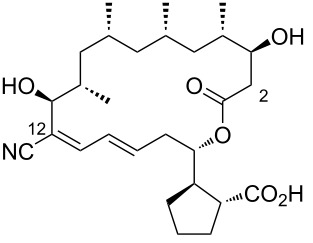 **9**	*Streptomyces rochei* SCSIO ZJ89	[22]
10	borrelidin G	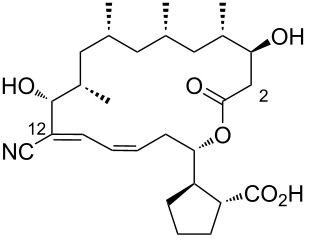 **10**	*Streptomyces rochei* SCSIO ZJ89	[22]
11	borrelidin H	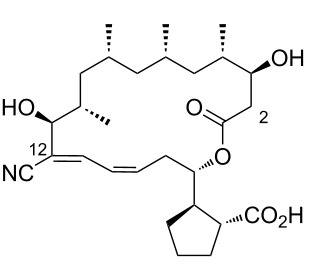 **11**	*Streptomyces rochei* SCSIO ZJ89	[22]
12	borrelidin I	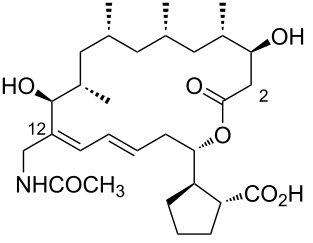 **12**	*Streptomyces rochei* SCSIO ZJ89	[22]
13	borrelidin J	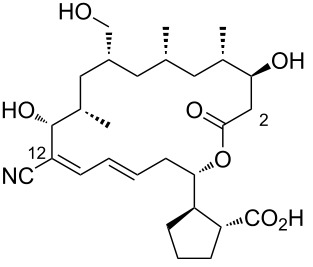 **13**	an endophytic *Streptomyces* sp. NA06554 from *Aster tataricus* collected in Aba County of Sichuan Province, China.	[23]
14	borrelidin K	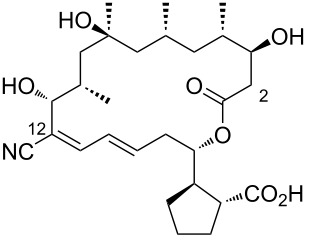 **14**	an endophytic *Streptomyces* sp. NA06554. from *Aster tataricus* collected in Aba County of Sichuan Province, China. marine pulmonated mollusks *Onchidium sp.* associated *Streptomyces olivaceus* SCSIO LO13 (Daya Bay, South China Sea)	[21,23]
15	borrelidin L	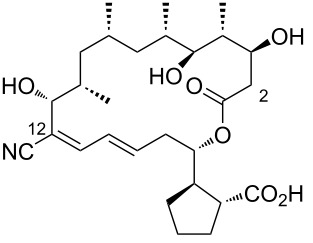 **15**	an endophytic *Streptomyces* sp. NA06554from *Aster tataricus*	[23]
16	borrelidin M	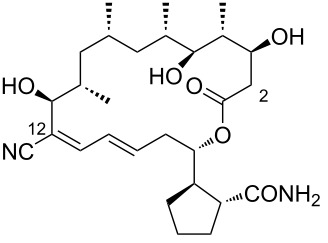 **16**	marine pulmonated mollusks *Onchidium sp.* associated *Streptomyces olivaceus* SCSIO LO13 (Daya Bay, South China Sea)	[21]
17	borrelidin N	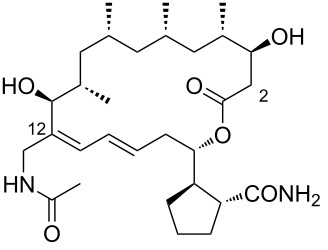 **17**	marine pulmonated mollusks *Onchidium sp.* associated *Streptomyces olivaceus* SCSIO LO13 (Daya Bay, South China Sea)	[21]
18	borrelidin O	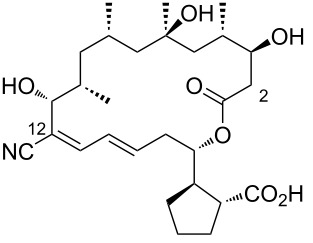 **18**	marine pulmonated mollusks *Onchidium sp.* associated *Streptomyces olivaceus* SCSIO LO13 (Daya Bay, South China Sea)	[21]
19	12-desnitrile-12- carboxyborrelidin	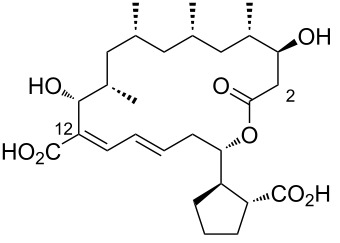 **19**	an endophytic *Streptomyces* sp. NA06554. from *Aster tataricus*	[23]

There are five total syntheses of borrelidin in the literature, reported by Morken et al. (2003) [25], Hanessian et al. (2003) [26], Ōmura et al. (2004) [27], Theodorakis et al. (2004) [28], and Ōmura et al. (2007) [29]. Additionally, there are twelve synthetic studies toward borrelidin reported by Haddad et al. (1997) [30], Haddad et al. (1997) [31], Theodorakis et al. (2003) [32], Herber and Breit (2006) [33], Iqbal et al. (2006) [34], Iqbal et al. (2008) [35], Yadav et al. (2009) [36], Minnaard and Madduri (2010) [37], Laschat et al. (2011) [38], Yadav and Yadav (2013) [39], Zhou et al. (2018) [40], and Uguen and Gembus (2019) [41], with seven focusing on strategies to access key fragments of borrelidin, including Ōmura’s C3–C11 fragment [23], Theodorakis’ C3–C11 fragment [33,38], Morken’s C1–C11 fragment [25], and Ōmura’s C1–C11 fragment [36–37,39]. Moreover, several derivatives have been synthesized by Moss et al. (2006) [42], Wilkinson et al. (2006) [43], Sunazuka et al. (2013) [44], Hahn et al. (2014) [45], Laschat et al. (2016) [46], and Huang et al. (2018) [47]. This demonstrates that borrelidin, with its remarkable biological activities and complex structure, remains an attractive target for synthetic organic chemists worldwide.

Several comprehensive reviews on borrelidin have been published, with a strong focus on its synthesis. The first notable comparison of total synthesis methods was conducted by Ōmura in 2005 [48], highlighting four pioneering approaches: Morken (2003), Hanessian (2003), Ōmura (2004), and Theodorakis (2004). This review also included two synthetic studies by Haddad [30–31] and Negishi [49]. In 2011, Darna et al. expanded this scope by incorporating Omura’s 2007 method along with biosynthesis studies and investigations into fragment synthesis, providing a more holistic perspective on borrelidin’s chemical synthesis and biological relevance [50]. However, in-depth works focusing on key fragment optimizations remain limited. Hence, this review aims to address this gap.

## Review

### Syntheses of borrelidin fragments

#### Uguen’s approach for constructing Morken’s C2–C12 fragment

**Scheme 1:**
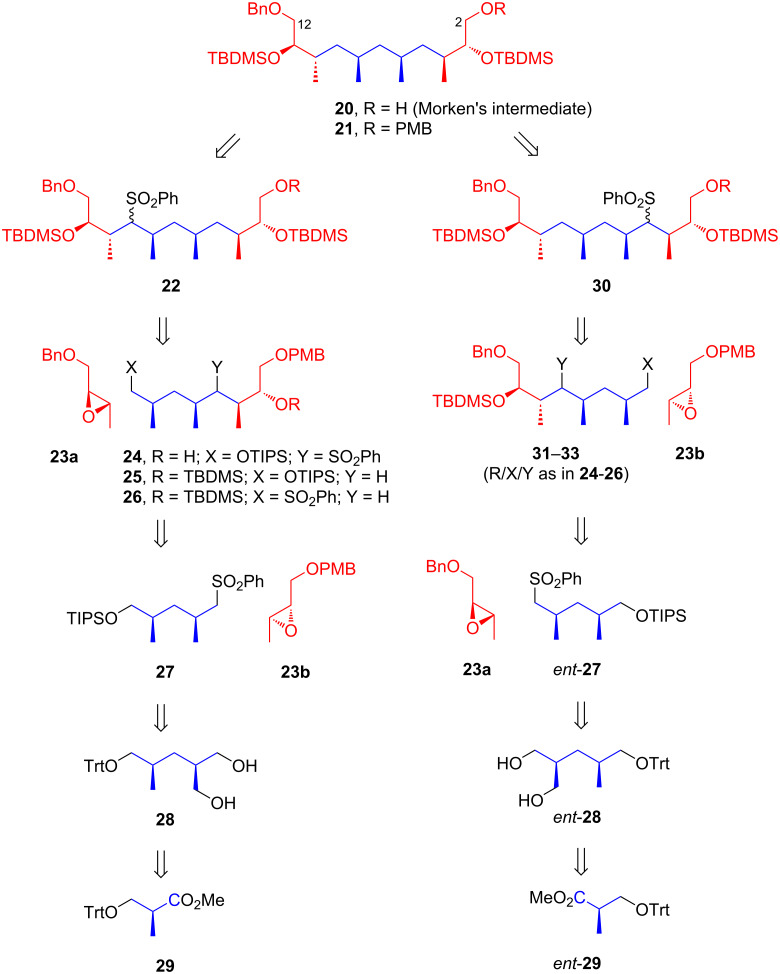
Synthetic strategy for Morken’s C2–C12 intermediate **20** as reported by Uguen et al. [41].

In 2019, Uguen and co-workers introduced a strategy to assemble Morken’s C2–C12 intermediate **20** [41]. Their approach utilized iterative base-catalyzed condensation of sulfone compounds with epoxides. As illustrated in Scheme 1, the monoalcohol **20** was prepared via PMB removal of compound **21**, which, in turn, was obtained through desulfonylation of compound **22**. Compound **22** originated from the condensation of epoxide **23a** with sulfone **26**, which was produced by desilylation of **25** followed by converting the resulting primary alcohol into a sulfone group. Intermediate **25** was prepared through TBDMS protection and desulfonylation of **24**, itself derived from the condensation of epoxide **23b** and sulfone **27**. The precursor **27** was synthesized from Roche ester **29** via a sequence of steps, including reduction, three-carbon homologation, and enzymatic desymmetrization. An alternative route was also proposed for synthesizing **21** from compound **30**, which was derived from *ent*-**29**. Notably, epoxides **23a** and **23b** were obtained via Sharpless epoxidation of (*E*)-2-butenol.

**Scheme 2:**
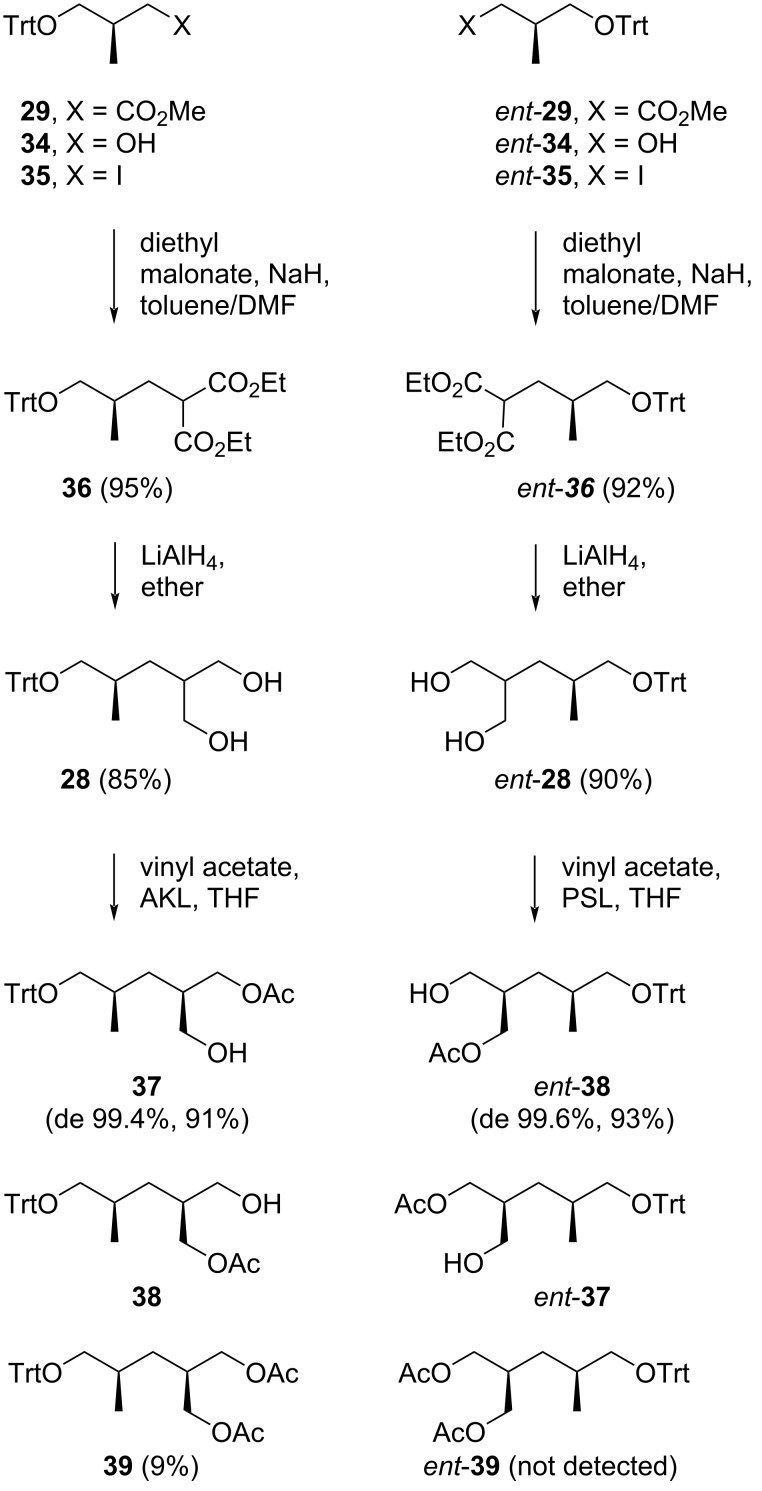
Preparation of monoacetates **37** and *ent*-**38** by Uguen et al. [41].

Uguen and co-workers began their synthesis by reducing Roche esters **29** and *ent*-**29** to their respective primary alcohols, **34** and *ent*-**34**, after recrystallization from hot hexane (100% ee by chiral phase HPLC, yield not reported). These alcohols were then treated with triphenylphosphine and iodine in the presence of imidazole to yield the iodides **35** and *ent*-**35** (Scheme 2). The iodide intermediates were subsequently reacted with deprotonated diethyl malonate to obtain compounds **36** and *ent*-**36** in 95 and 92% yield, respectively, over two steps from **34** and *ent*-**34**. The diols **28** (85%) and *ent*-**28** (90%) were isolated after reduction of their parent malonates **36** and *ent*-**36** using LiAlH_4_ in ether. Following several experiments with vinyl acetate as the acylating agent, Amano lipase AK (AKL) was identified as the most effective biocatalyst for achieving selective acetylation, converting diol **28** to the monoacetate **37** in 91% yield with >99.4% de (by HPLC). The diacetate byproduct **39** was formed in a small amount (9%). A similar diol desymmetrization of *ent*-**28** was best achieved with Amano lipase PS (PSL), yielding monoacetate *ent*-**37** (de 99.6%, 93% yield) without the formation of diacetate *ent*-**39**.

**Scheme 3:**
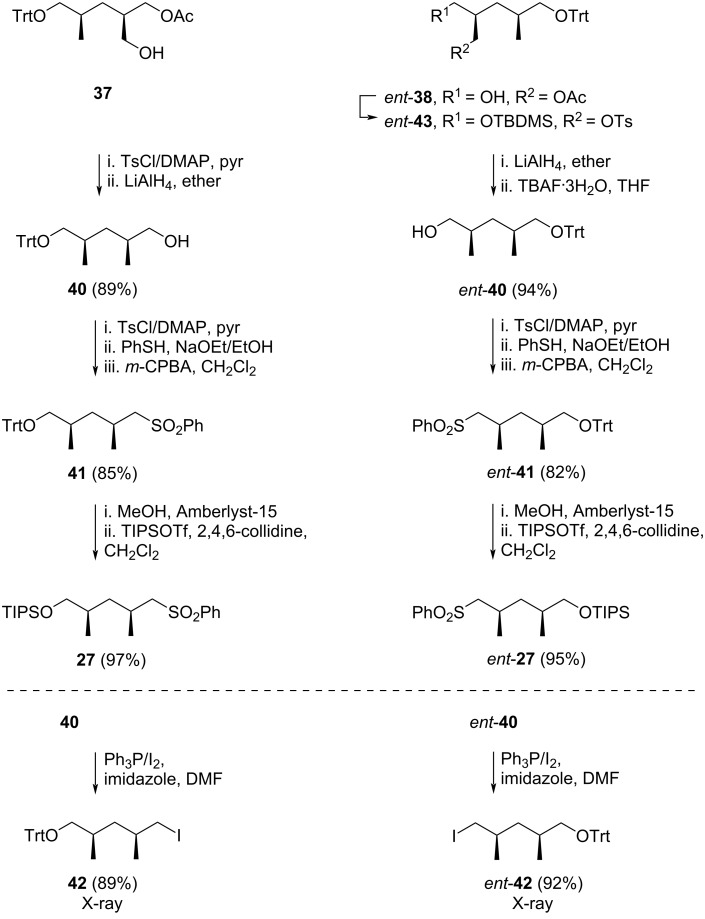
Preparation of sulfones **27** and *ent*-**27** by Uguen et al. [41].

Compound **37** was tosylated using tosyl chloride in pyridine with the addition of DMAP and the resulting product was treated with LiAlH_4_ in ether to form alcohol **40** (89%) (Scheme 3). The primary alcohol **40** was then converted to its iodide derivative **42** (89%), from which single crystals were obtained, and its structure was determined unequivocally by XRD crystallography. Iodide **42** was then reacted with sodium phenylsulfinate in DMF to afford the corresponding sulfone **41**. However, Uguen found that a more efficient route involved treating alcohol **40** with tosyl chloride in pyridine and DMAP, followed by nucleophilic displacement with sodium thiophenol and oxidation of the resulting sulfide with *m*-CPBA, yielding sulfone **41** in 85% yield over three steps. This compound was isolated in its pure form as a white solid after recrystallization from ethanol, confirmed by HPLC and NMR. In parallel, the primary alcohol group of *ent*-**38** was protected as a TBDMS ether, and the acetate group was converted to a tosyl ester by hydrolyzing the acetate functionality using potassium carbonate in methanol, followed by reaction with tosyl chloride in pyridine and DMAP. The product, *ent*-**43**, was obtained in 79.5% yield over three steps. This compound was reduced using LiAlH_4_ and treated with TBAF to remove the TBDMS group, yielding alcohol *ent*-**40** in 94% yield. Like **40**, iodination of *ent*-**40** gave crystalline product *ent*-**42**, and its structure was confirmed by XRD crystallography. The intermediate *ent*-**40** was then subjected to the same tosylation/thiolation/oxidation sequence used for the **40** to **41** conversion, yielding *ent*-**41** in comparable yield (82%). Treatment of both **41** and *ent*-**41** with acidic resin (Amberlyst-15), followed by protection of the resultant primary alcohol with TIPSOTf in the presence of 2,4,6-collidine provided the anticipated compounds **27** (97%) and *ent*-**27** (95%).

**Scheme 4:**
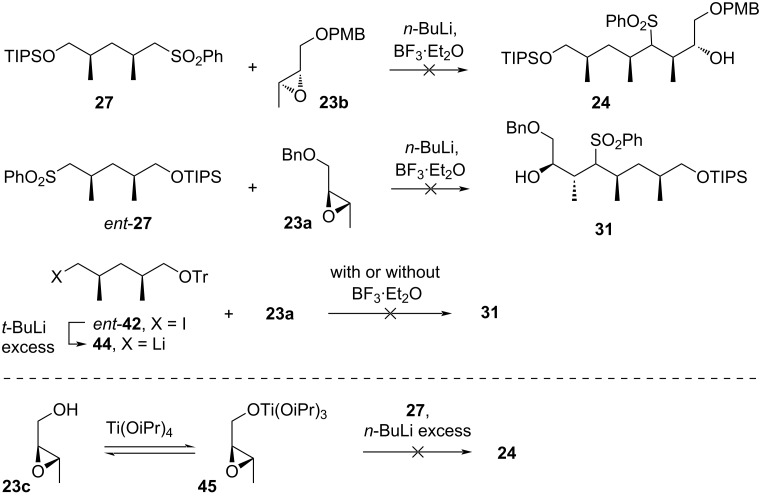
Attempts to couple sulfones **27** and *ent*-**27** with epoxides **23a**–**c** reported by Uguen et al. [41].

With **27** and *ent*-**27** in hand, the next step was to perform the coupling of these sulfones with epoxides **23b** and **23a**, respectively. Following the literature procedure for a similar reaction, using *n*-butyllithium in the presence of BF_3_·Et_2_O at −78 °C, the coupling reaction unfortunately resulted in the decomposition of the reactants (Scheme 4). The authors hypothesized that the failure may have been due to the low reactivity of sulfones **27** and *ent*-**27** under the reaction conditions. Additionally, treating *ent*-**42** with excess *tert*-butyllithium to form the corresponding lithiated derivative and reacting it with epoxide **23a**, both with or without BF_3_·OEt_2_, also led to unsatisfying results, with reactant decomposition observed. Similarly, when the lithium derivative was reacted with CuSPh to form the corresponding heterocuprate species prior to reacting with the epoxide, the same decomposition occurred, despite this strategy being successful in a related case. Finally, replacing the epoxides **23b** and **23a** with the non-protected variant **23c**, and reacting it with sulfone **27** after pre-complexation with Ti(OiPr)_4_, again led only to decomposition.

**Scheme 5:**
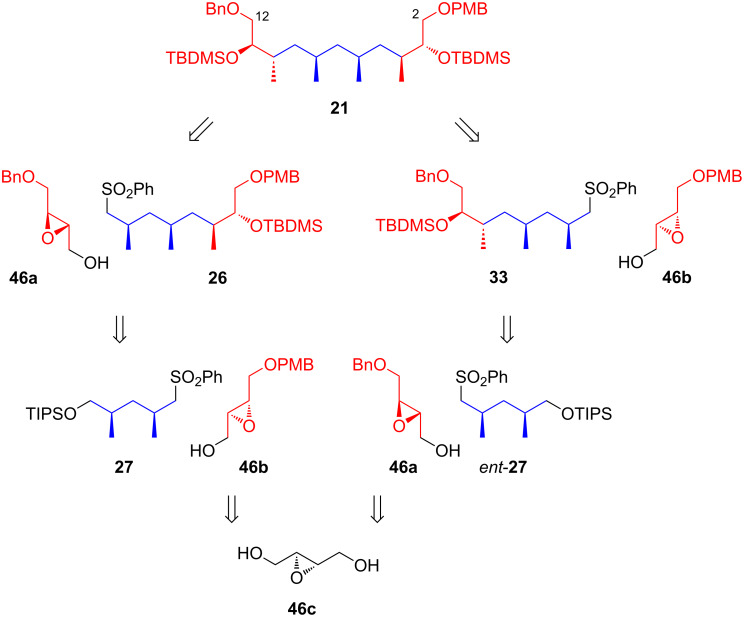
Modified synthetic plan for Morken’s C2–C12 intermediate by Uguen [41].

**Scheme 6:**
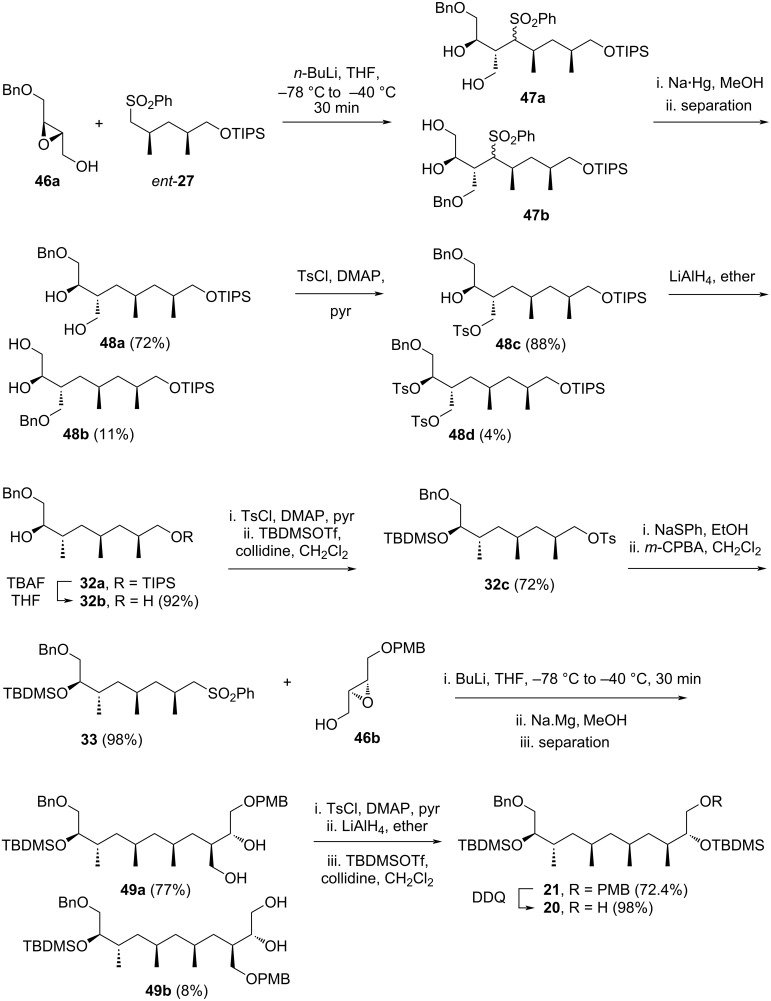
Revised synthetic strategy for Morken’s C2–C12 intermediate **20** by Uguen [41].

Given the unsatisfying results, Uguen and co-workers replaced the epoxides **23a**–**c** to monoethers **46a** and **46b**, derived from *trans*-2,3-epoxy-1,4-butanediol **46c**, and revised their synthetic strategy as shown in Scheme 5. The synthesis was restarted by treating the pre-cooled (−78 °C) THF mixture of sulfone *ent*-**27** and epoxide **46a** with *n*-butyllithium in a molar ratio of 1.5:1:2.5 (Scheme 6). After warming the mixture for 15 minutes to approximately −40 °C and leaving it at the same temperature for an additional 15 minutes, two products, **47a** and **47b**, were formed. After desulfonylation with sodium amalgam (Na·Hg) in methanol and column chromatographic purification, the anticipated diols **48a** and **48b** were obtained in 72 and 11% yield, respectively. The authors noted that using freshly prepared sodium amalgam (within two hours) was critical for achieving a good yield. Compound **48a** was then tosylated using TsCl/DMAP/pyridine to give monotosylate **48c** in 88% yield, with a small amount (4%) of the undesired ditosyl product **48d**. The desired compound **48c** was reduced with LiAlH_4_ to remove the OTs group, and after silyl group removal, diol **32b** was obtained in 92% yield. Protection of the primary alcohol as a tosyl ester and the secondary as a TBDMS ether afforded intermediate **32c** (72%). This intermediate was then treated with sodium thiophenol in ethanol, followed by oxidation with *m*-CPBA to provide sulfone **33**, which readily was reacted with another epoxide **46b**. The sequential treatments used for the condensation of *ent*-**27** and **46a** to **48a** were applied to **33** and **46b**, yielding the desired product **49a** (77%) along with a small amount of isomeric **49b** (8%). The primary alcohol of **49a** was tosylated and then reduced with LiAlH_4_, while the secondary alcohol group was protected as a silyl ether using TBDMSOTf and collidine. The resulting product, **21**, was isolated in 72.4% yield over three steps. Finally, treatment with DDQ afforded the target Morken’s C2–C12 intermediate **20** in 98% yield.

The authors highlighted that shifting the epoxides from **23a**,**b** to **46a**,**b** resulted in the additional tosylation/reduction sequence to install the C4 and C10 methyl moieties of the desired target molecule, making the overall synthesis more lengthy. Thus, **20** was obtained from *ent*-**29** over 25 steps in 11% overall yield. On the other hand, the efficiency of the process was emphasized by the authors on the utilization of Roche esters **29** and *ent*-**29** to prepare stereochemically pure sulfones **27** and *ent*-**27**, respectively. Moreover, the use of the trityl protecting group facilitated a simple impurity removal from the key chiral intermediates through recrystallization, from which their exact structures could be elucidated by XRD crystallography.

#### Zhou’s approach for constructing Omura’s C3–C11 fragment

In 2018, Zhou and co-workers developed an efficient, high-yielding iterative synthesis of polydeoxypropionate based on iridium-catalyzed asymmetric hydrogenation of α-substituted acrylic acid [40]. This method was subsequently applied to the synthesis of a promising vaccine candidate (+)-phthioceranic acid, as well as key intermediates for two natural products, ionomycin and borrelidin (C3–C11). The synthesis involved three main steps: (1) carboxymethylation using Meldrum’s acid, (2) alkenylation with Eschenmoser’s salt, and (3) asymmetric hydrogenation catalyzed by iridium complex (*R_a_*)-**50** or (*S_a_*)-**50**.

**Scheme 7:**
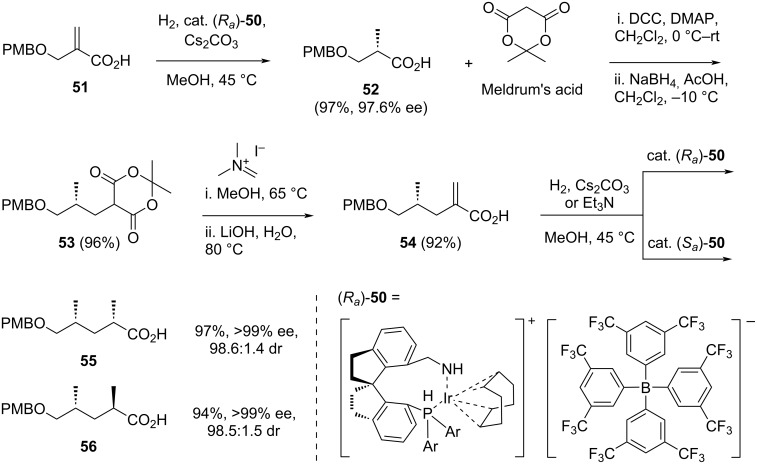
Iterative synthesis of polydeoxypropionates developed by Zhou et al. [40].

The authors began their investigation by performing the hydrogenation of α-substituted acrylic acid **51** (Scheme 7). After optimization, the iridium complex (*R_a_*)-**50**, in the presence of cesium carbonate, was identified as the most efficient catalyst, producing compound **52** in 97% yield with an enantiomeric excess of 97.6%. Subsequently, compound **52** was treated with Meldrum’s acid in the presence of DCC and DMAP. The reaction was allowed to proceed for 6 h at room temperature, after which the temperature was reduced to −10 °C, and sodium borohydride was added. The mixture was left to react for 12 hours, yielding compound **53** in 96%. This intermediate was then converted to acrylic acid **54** by reacting it with Eschenmoser’s salt, followed by hydrolysis with lithium hydroxide. The resulting unsaturated acid **54**, isolated in 92% yield, underwent asymmetric hydrogenation using both (*R_a_*)-**50** and (*S_a_*)-**50** catalysts. This step provided the respective compounds **55** and **56** in excellent high yield and stereoselectivity. The authors emphasized that, as the alkene moiety in **54** was in a terminal position, the stereoselectivity of the products was determined solely by the chiral environment of the catalysts.

**Scheme 8:**
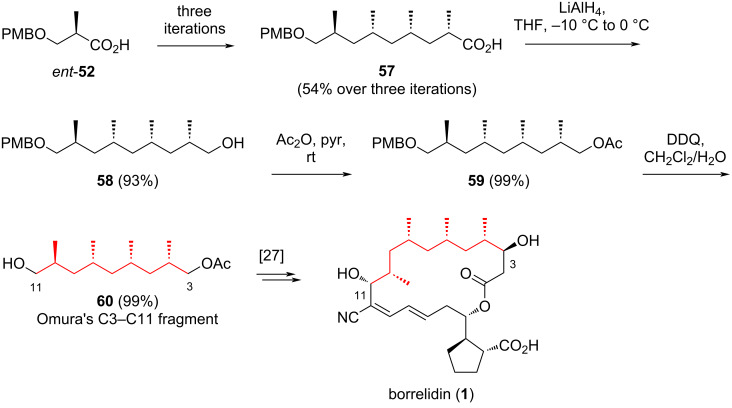
Application of iterative synthesis of polydeoxypropionate to construct the C3–C11 fragment **60** of borrelidin **1**.

The application of this method to construct the C3–C11 fragment **60** of borrelidin is summarized in Scheme 8. Starting from *ent*-**52**, obtained via the asymmetric hydrogenation of **51** using the catalyst (*S_a_*)-**50**, the previously developed three-steps reaction sequence was adopted and repeated three times, yielding polydeoxypropionic acid **57** in an overall yield of 54%. The iridium catalyst (*R_a_*)-**50** was chosen to ensure the correct stereochemistry of the newly formed three stereocenters. Additionally, replacing cesium carbonate with triethylamine proved crucial for achieving efficient asymmetric hydrogenation in this case. Subsequently, the carboxylic acid group of **57** was reduced with LiAlH_4_ in THF to produce primary alcohol **58** in 93% yield. This alcohol was then acetylated using acetic anhydride and pyridine reagent. Finally, the resulting acetate **59** was treated with DDQ, affording the target compound **60** in 99% yield, corresponding to an overall yield of 49% over 18 steps starting from **51**.

#### Yadav’s approach for constructing Omura’s C1–C11 fragment

**Scheme 9:**
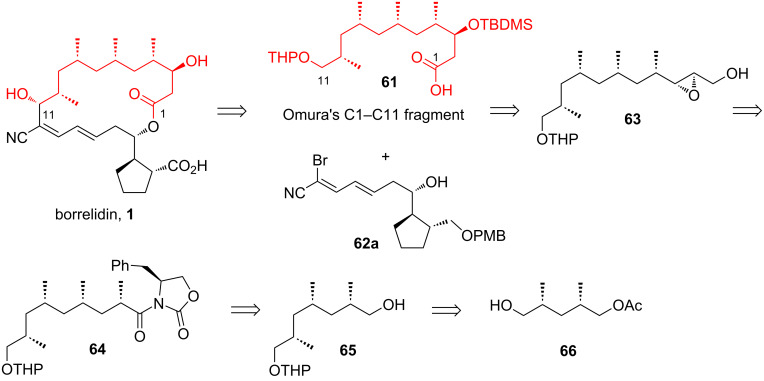
Retrosynthetic analysis of borrelidin by Yadav et al. [39].

Yadav and Yadav, in 2013, reported their work on preparing the C1–C11 fragment **61** of borrelidin. Their approach employed an iterative sequence of oxidation, Wittig olefination, hydrogenation, and asymmetric methylation for carbon homologation, alongside Sharpless epoxidation and regioselective reduction to install the hydroxy group at the C3 position [39]. In their retrosynthetic analysis, the target molecule **61** was envisioned to be obtained from epoxide **63** through regioselective opening of the epoxide ring, oxidation of the resulting primary alcohol to a carboxylic acid, and protection of the secondary alcohol as a TBDMS ether (Scheme 9). Intermediate **63** was planned to be derived from Evans’ amide **64** by reducing the amide moiety to a primary alcohol, oxidizing it to an aldehyde, performing a Wittig olefination to install an unsaturated ester, reducing the ester to a primary alcohol, and then conducting asymmetric epoxidation of the double bond. Evan’s amide **64** would be synthesized from primary alcohol **65** through a sequence of oxidation to aldehyde, Wittig olefination to an unsaturated ester, hydrogenation of the olefin, conversion of the ester to Evans’ amide, and asymmetric methylation. Intermediate **65**, in turn, would be obtained from the known five-carbon precursor **66** through the iterative sequence of oxidation, Wittig olefination, hydrogenation, asymmetric methylation, followed by reduction of the carboxyl group and protection of the resulting alcohol as a THP ether.

**Scheme 10:**
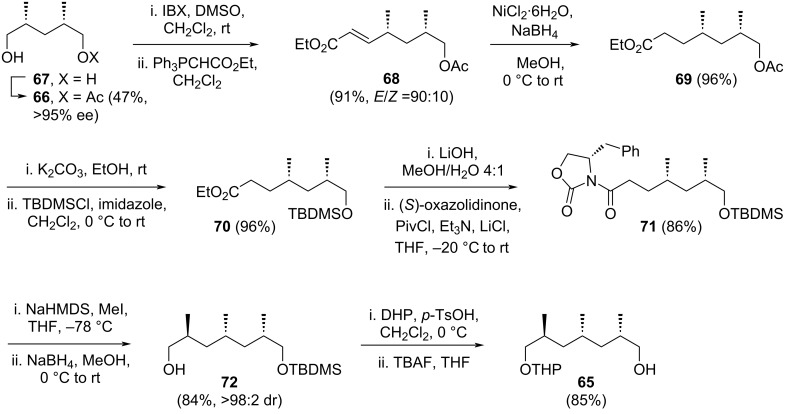
Two-carbon homologation of precursor **66** in the synthesize C1–C11 fragment **61** of borrelidin [39].

Yadav and Yadav commenced their synthesis with the enzymatic desymmetrization of *meso-*diol **67** to monoacetate **66**, achieving a 47% yield with an enantiomeric excess greater than 95%, using porcine pancreatic lipase (PPL) and vinyl acetate in THF (Scheme 10). The remaining primary alcohol in **66** was oxidized to its corresponding aldehyde using IBX. Subsequent the two-carbon elongation of this aldehyde yielded unsaturated ester **68** in 91% yield with an *E*/*Z* ratio of 90:10. The double bond in ester **68** was reduced using sodium borohydride in the presence of NiCl_2_·6H_2_O, affording ester **69** in 96% yield. Hydrolysis of the acetate group in **69** with potassium carbonate followed by treatment with TBDMSCl and imidazole converted it into silyl ether **70**. The ester group in **70** was then hydrolyzed using lithium hydroxide, and the resulting acid was coupled with Evans’ chiral oxazolidinone in the presence of pivaloyl chloride, triethylamine, and lithium chloride to produce compound **71** in 86% yield. Diastereoselective methylation of **71** was achieved by treating it with NaHMDS at low temperature, followed by the addition of methyl iodide, resulting in a diastereomeric ratio greater than 98:2. Reduction of the product to remove the Evans auxiliary furnished primary alcohol **72** in 84% yield. This alcohol was then protected as a THP ether, and the TBDMS group was removed using a fluoride source, yielding another primary alcohol **65** in 85% yield, thus completing the synthesis of the left-hand portion of the target molecule.

**Scheme 11:**
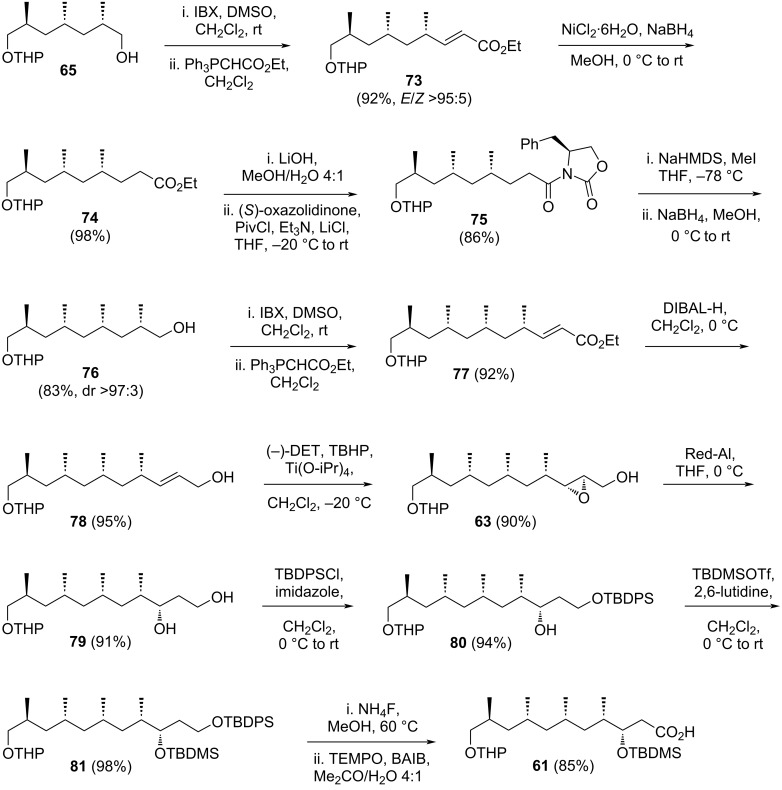
Synthesis of the C1–C11 fragment **61** of borrelidin from monoalcohol **65** [39].

Another series of oxidation, Wittig olefination, reduction, and asymmetric methylation was applied to the right-hand side of compound **65**, affording compound **76**. This transformation added two carbons to the backbone and introduced a methyl branch with the correct stereochemistry (Scheme 11). A further two-carbon homologation of compound **76** through an oxidation and Wittig olefination sequence yielded unsaturated ester **77** in 92% yield. The ester group in **77** was reduced using DIBAL-H, achieving a 95% yield. Sharpless epoxidation of alcohol **78** was then performed using (−)-DET, *tert*-butyl hydroperoxide (TBHP), and Ti(OiPr)_4_, resulting in the desired epoxide **63** with a 90% yield. Regioselective reductive opening of this epoxide was successfully carried out with Red-Al^®^, yielding diol **79**. The more reactive primary alcohol in diol **79** was selectively masked as TBDPS ether **80** (94%), followed by protection of the secondary alcohol as TBDMS ether **81** (98%). The primary alcohol was then liberated using ammonium fluoride in hot methanol (60 °C). Oxidation of this alcohol to a carboxylic acid was achieved using TEMPO and (diacetoxyiodo)benzene (BAIB), completing the synthesis of the target molecule **61**. The final compound **61** was obtained in 18.4% overall yield over 27 steps starting from precursor **66**.

#### Laschat’s strategy for constructing Theodorakis’ C3–C11 fragment

Laschat and co-workers, in 2011, developed a chemoenzymatic strategy for synthesizing Theodorakis’ C3–C11 fragment **82** of borrelidin [38]. They highlighted a significant limitation in existing methods for constructing the deoxypropionate unit of borrelidin, which features four 1,3-alternating methyl groups with a *syn,syn,anti*-configuration. These methods typically required at least three synthetic steps to iteratively form each stereocenter, significantly reducing overall efficiency. To overcome this challenge, Laschat’s approach leveraged a chiral pool building block – methyl-branched preen gland wax ester – as the starting material. This ester already contained three methyl groups pre-installed with the stereochemistry necessary for borrelidin, streamlining the synthesis process.

**Scheme 12:**
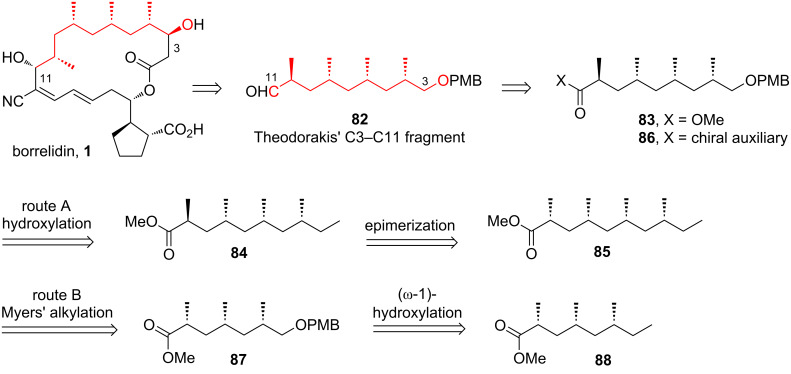
Synthetic plan for Theodorakis’ C3–C11 fragment **82** of borrelidin by Laschat et al. [38].

The retrosynthetic analysis by Laschat and co-workers is described in Scheme 12. The target molecule **82** was envisioned to be derived from esters **83** or **86**, depending on the chosen synthetic pathway. In route A, ester **83** was designed to originate from compound **84** through a series of sequential steps, including chemoenzymatic (ω-1)-hydroxylation, regioselective dehydration of the resulting alcohol to form a terminal alkene, ozonolysis of the alkene to yield an aldehyde, reduction of the aldehyde product to a primary alcohol, and protection of the alcohol as a PMB ether. Compound **84** could be obtained from the all-*syn* isomer **85** through an epimerization process. In route B, compound **86** was proposed to be synthesized from methyl ester **87**. The transformation involved reduction of **87** to a primary alcohol, conversion of the alcohol into the corresponding iodide, and subsequent nucleophilic substitution with deprotonated (*R*,*R*)-**91**. The introduction of the OPMB functionality in compound **88** could then be achieved by following the steps employed in the transformation of **84** to **83**.

**Scheme 13:**
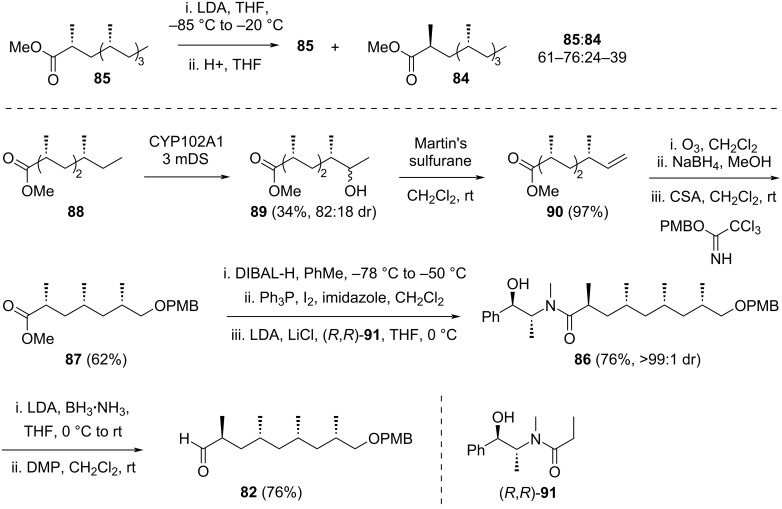
Synthesis of Theodorakis’ C3–C11 fragment **82** from compound **88** [38].

The synthesis via route A began with efforts to optimize the epimerization of compound **85** to produce **84** (Scheme 13). After numerous attempts, it was found that treating **85** with LDA at low temperature followed by the addition of various acids yielded **84** as the minor product, with the product ratio of **85**/**84** ranging from 2:1 to 3:1. Attempts to influence the stereochemical outcome by employing chiral proton sources, such as ᴅ- and ʟ-menthol, (+)- and (–)-camphorsulfonic acid, or pseudoephedrinamide (*R*,*R*)-**91**, proved unsuccessful, as they did not significantly alter the stereochemical preference. Additionally, reversing the quenching order by adding the enolates to acids also failed to impact the outcome. Subsequently, separation of **84** from **85** was explored using various lipases and an esterase. However, preliminary experiments with these enzymes did not yield satisfactory results.

The focus then shifted to route B, utilizing methyl 2,4,6-trimethyloctanoate **88** as the starting material. Hydroxylation at the (ω-1) position was achieved using the NADH-dependent mutated enzyme variant CYP102A1 3 mDS, a p450 monooxygenase derived from *Bacillus megaterium* CYP102A1. After chromatographic purification, alcohol **89** was obtained in a 34% yield with a diastereomeric ratio of 82:18. This alcohol was subsequently dehydrated using Martin’s sulfurane to produce terminal alkene **90** in 97% yield. A sequence of ozonolysis, reduction with sodium borohydride, and PMB protection using camphorsulfonic acid and PMB-trichloroacetimidate reagents followed, yielding compound **87** in 62% yield. Next, the ester functionality of **87** was reduced to the corresponding primary alcohol with DIBAL-H and converted to the iodide derivative using Ph_3_P/I_2_/imidazole reagents. The resulting iodide was treated with lithiated (*R*,*R*)-**91** to afford compound **86** in a 76% yield with excellent diastereoselectivity (>99:1 dr). Finally, reductive cleavage of the chiral auxiliary using a combination of LDA and BH_3_·NH_3_ provided the target molecule **82** in a 76% yield. The authors emphasized that the Theodorakis’ C3–C11 fragment **82** of borrelidin was synthesized via a concise 8-step route, achieving a 36% overall yield from the chiral pool precursor **88**.

#### Minnaard’s strategy for constructing Ōmura’s C1–C11 and C12–C23 fragments

**Scheme 14:**
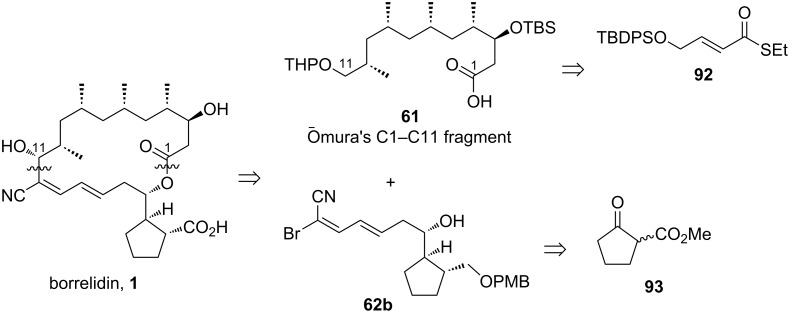
Retrosynthesis of **61** and **62b** by Minnaard and Madduri [37].

Minnaard and Madduri in 2010 [37] developed a novel strategy to prepare the C1–C11 and C12–C23 fragments of borrelidin, representing the upper and lower parts of this natural product as classified by Ōmura [27,29]. Their method was based on the concept of “catalytic total synthesis”, wherein all stereocenters were installed under the control of catalysts. Minnaard and Madduri proposed the synthesis of the C1–C11 fragment from unsaturated thioester **92** through iterative, previously developed asymmetric 1,4-addition reactions (key step), reduction of the thioester moiety to an aldehyde, and olefination to produce another unsaturated ester (Scheme 14). In contrast, the synthesis of the lower part, the C12–C23 fragment, was designed to proceed from ester **93** via asymmetric hydrogenation (key step), sequential protection and deprotection steps, functional group transformations, stereocontrolled allylation, cross-metathesis, and Horner–Wadsworth–Emmons (HWE) olefination. This method highlights the power of catalytic stereocontrol, achieving the complex architecture of borrelidin fragments with efficiency and precision.

**Scheme 15:**
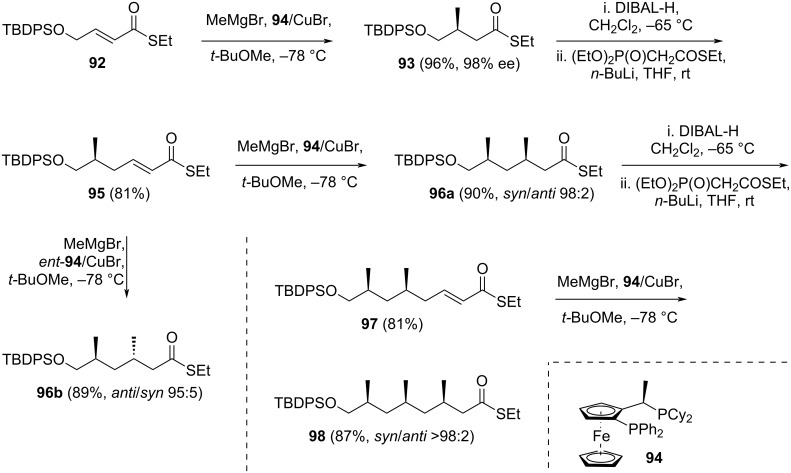
Synthesis of intermediate **98** by Minnaard and Madduri [37].

The synthesis commenced with the treatment of precursor **92**, obtained in three steps from ethylene glycol, with MeMgBr and catalytic **94**/CuBr, yielding the 1,4-addition product **93** in 96% yield and excellent regioselectivity (ee 98%) (Scheme 15). The authors highlighted the scalability of this reaction, successfully processing up to 15 g of starting material. Compound **93** was reduced to its corresponding aldehyde using DIBAL-H, followed by Horner–Wadsworth–Emmons (HWE) olefination with (EtO)_2_P(O)CH_2_COSEt, resulting in the unsaturated thioester **95**. Reapplying the 1,4-addition reaction conditions to **95** produced the *syn*-product **96a** in 90% yield with a diastereomeric ratio of 98:2. Interestingly, substituting the catalyst with *ent*-**94** delivered **96b** in 89% yield and dr 95:5. Subsequently, **96a** was subjected to a similar sequence of reduction, HWE olefination, asymmetric 1,4-addition, culminating in compound **98** in 70% overall yield across three steps, with a dr exceeding 98:2.

**Scheme 16:**
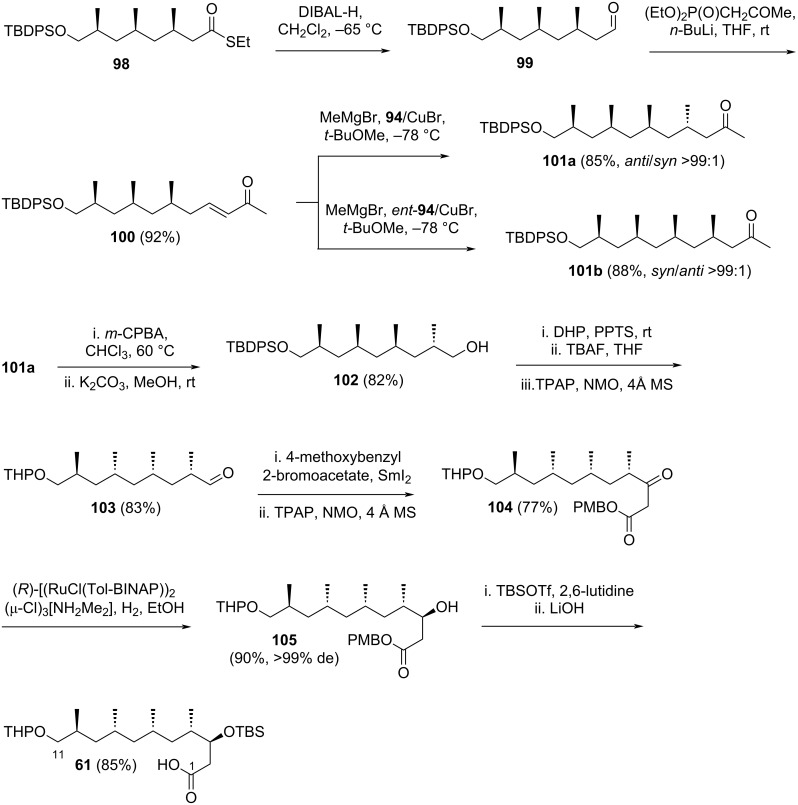
Synthesis of Ōmura’s C1–C11 fragment **61** by Minnaard and Madduri [37].

The reaction continued with the reduction of thioester **98** to aldehyde **99**, followed by HWE olefination with (EtO)_2_P(O)CH_2_COMe, yielding compound **100** in 92% yield (Scheme 16). The stereocontrol achieved by the catalysts was again demonstrated when compound **100** was treated with MeMgBr/CuBr and either **94** or *ent*-**94**, affording **101a** and **101b**, respectively, with high yield and exclusive diastereoselectivity. Compound **101a**, featuring the relevant stereochemistry of borrelidin at the C4, C6, C8, and C10, underwent Baeyer–Villiger oxidation using *m*-CPBA. Subsequent hydrolysis of the resulting ester with K_2_CO_3_ in methanol provided alcohol **102** in 82% yield. The free primary alcohol of **102** was protected as a THP ether, and the TBDPS group was removed to expose the opposite free primary alcohol, which was oxidized to aldehyde **103** in 83% yield over three steps using TPAP/NMO reagents. Compound **103** was subjected to a SmI_2_-mediated Reformatsky-type reaction with 4-methoxybenzyl 2-bromoacetate, followed by oxidation of the resulting β-hydroxy intermediate with TPAP/NMO, producing keto ester **104** in 77% yield over two steps. Catalytic asymmetric hydrogenation of **104**, employing (*R*)-[(RuCl(Tol-BINAP))_2_(μ-Cl)_3_[NH_2_Me_2_], yielded the product in 90% yield with nearly complete diastereoselectivity (de >99%). Finally, the secondary alcohol was protected as a TBS ether, and the ester group was hydrolyzed to deliver Ōmura’s C1–C11 fragment **61** in 85% yield.

**Scheme 17:**
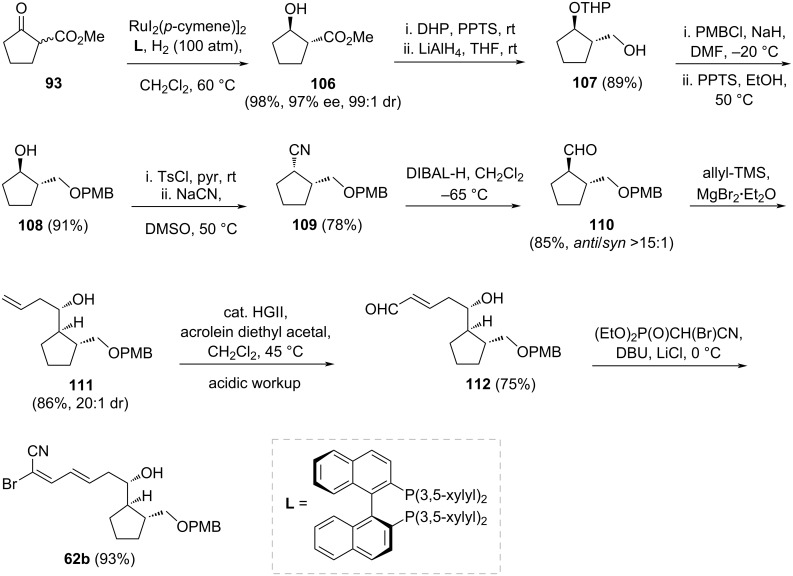
Synthesis of fragment **62b** of borrelidin as proposed by Minnaard and Madduri [37].

The synthesis of part **62b** began with the asymmetric hydrogenation of **93** to yield β-hydroxy ester **106** (Scheme 17). Initial experiments, following the procedure of Noyori et al. [51] and using [RuCl_2_(*p*-cymene)]_2_ metal complex with BINAP as the chiral ligand, produced **106** in 92% yield (92% ee, 99:1 dr). Optimized conditions were achieved by employing [RuI_2_(*p*-cymene)]_2_ with the chiral ligand 3,5-xylyl-BINAP, resulting in **106** with an improved yield of 98% (97% ee, 99:1 dr). The secondary alcohol of **106** was protected as a THP ether, and the ester group was reduced to a primary alcohol **107** in 89% yield. This primary alcohol was then protected as a PMB ether. After deprotecting the THP group, the resulting secondary alcohol was converted to a tosyl ester, which underwent an SN2 reaction with sodium cyanide in DMSO, yielding compound **109** with stereochemical inversion. Interestingly, reduction of the cyanide group with DIBAL-H to aldehyde **110** also resulted in stereochemical inversion (85%, *anti/syn* >15:1). Subsequent chelation-controlled allylation of aldehyde **110**, following Ōmura’s method [27,29], employed allyltrimethylsilane and MgBr_2_·OEt_2_, yielding allyl alcohol **111** in 86% yield with exclusive diastereoselectivity (20:1 dr). Direct cross-metathesis of **111** with acrolein was envisioned as an efficient method to introduce an aldehyde functionality adjacent to the alkene moiety without prior protection of the free allylic alcohol. This hypothesis was successfully realized by reacting **111** with acrolein diethyl acetal in the presence of Hoveyda–Grubbs’ second-generation catalyst, affording aldehyde **112** in 75% yield as the *E*-isomer after a careful acidic workup. Finally, HWE olefination of aldehyde **112** with (EtO)_2_P(O)CHBrCN completed the synthesis of fragment **62b** of borrelidin.

Minnaard and Madduri emphasized the significant role of asymmetric catalysis in their strategy, utilizing a copper-catalyzed asymmetric 1,4-addition and a ruthenium-catalyzed asymmetric ketone hydrogenation. Fragment **61** was synthesized in 15% overall yield across 19 steps, while fragment **62b** was achieved in 32% yield over 11 steps.

#### Herber and Breit’s strategy for constructing Theodorakis’ C3–C11 fragment

**Scheme 18:**
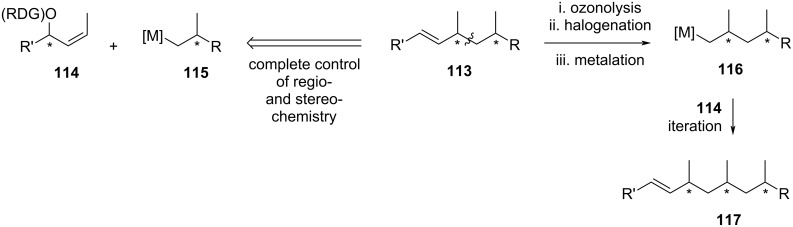
Iterative directed allylation for the synthesis of deoxypropionates by Herber and Breit [33].

In 2006, Herber and Breit utilized an iterative deoxypropionate synthesis to construct the Theodorakis’ C3–C11 fragment of borrelidin. This approach was based on a copper-mediated directed allylic substitution previously developed in their laboratory. The strategy primarily involved the reaction of a chiral organometallic reagent **115** with a chiral allyl electrophile **114**, as depicted in Scheme 18. The resulting deoxypropionate **113** was obtained with the newly formed stereocenter controlled by the reagent directing group (RDG) attached to the allyl precursor **114**. Iteration of this process required ozonolysis of **113**, followed by its conversion to an organometallic intermediate **116**, which was then reacted with allyl **114** to yield another deoxypropionate product, **117**.

**Scheme 19:**
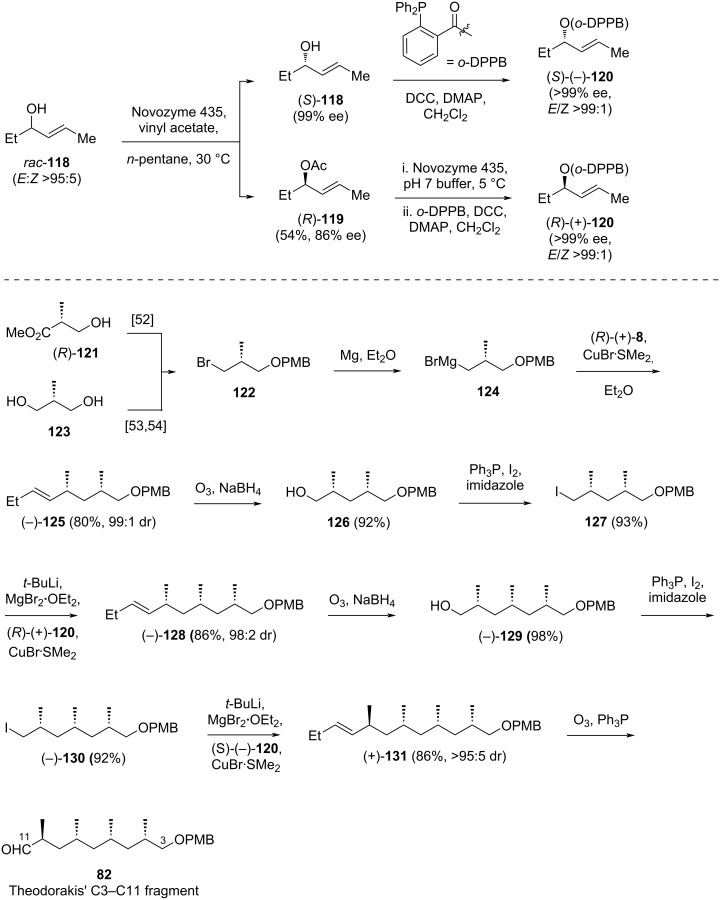
Iterative copper-mediated directed allyl substitution for the synthesis of Theodorakis’ C3–C11 fragment **82** developed by Herber and Breit [33].

The synthesis began with the preparation of the precursor chiral allyl ester (*R*)-**120** via enzymatic kinetic resolution of readily available *rac*-**118** using Novozyme 435 and vinyl acetate in pentane at 30 °C (Scheme 19). The reaction was halted at the conversion of 54%, yielding the remaining alcohol (*S*)-**118** with >99% ee. The product (*R*)-**119** was subsequently treated with Novozyme 435 in a pH 7 buffer to hydrolyze the acetate group. The resulting alcohol, (*R*)-**118**, was isolated in 73% yield with 96% ee. This alcohol was then reacted with *o*-diphenylphosphanylbenzoate (*o*-DPPB) in the presence of DCC, affording (*R*)-**120** in 83% yield (>99% ee, *E*/*Z* >99:1) after recrystallization. Similarly, the enantiomer (*S*)-**118** was esterified with *o*-DPPB under the same conditions, providing (*S*)-**120** in 88% yield (>99% ee, *E*/*Z* >99:1) after recrystallization. The authors noted that both (*R*)- and (*S*)-**120** could be stored in their crystalline forms for months without significant decomposition or undesired oxidation to the corresponding phosphane oxide.

The work continued with the preparation of the chiral organometallic reagent **124**. Starting from known bromide **122**, which was readily accessible from Roche ester (*R*)-**121** [52] or 1,3-diol **123** [53–54] through literature procedures, the Grignard reagent **124** was obtained by reaction with magnesium in anhydrous diethyl ether. Herber and Breit emphasized that activating the magnesium using a dry stirring method was crucial for smooth magnesiation. The freshly prepared Grignard reagent **124** was added to the allyl electrophile (*R*)-**120** in the presence of CuBr·SMe_2_, facilitating the formation of deoxypropionate **125** in 80% yield with excellent regioselectivity (>99:1) and stereoselectivity (99:1 dr). Ozonolysis of **125** followed by reductive work up with NaBH_4_ afforded alcohol **126** in 92% yield. At this stage, the authors explored an alternative procedure for preparing the organometallic reagent using halogen-metal exchange, which proved feasible for small scale operations. Alcohol **126** was converted into its iodide derivative **127** in 93% yield using Ph_3_P/I_2_/imidazole reagent. Halogen-metal exchange of **127** with *tert*-butyllithium proceeded efficiently, and subsequent transmetallation with MgBr_2_·OEt_2_ yielded the desired magnesium species. Reaction of this reagent with (*R*)-**120** in the presence of CuBr·SMe_2_ afforded product (–)-**128** in 86% yield, with perfect regioselectivity (>99:1) and excellent stereochemistry (98:2 dr). Repeating the sequence of ozonolysis, reductive work up, iodination, halogen-metal exchange, and transmetallation for compound (–)-**128**, followed by reaction with (*S*)-**120**, provided the deoxypropionate product (+)-**131** in 78% yield over three steps, with dr >95:5. Finally, another ozonolysis followed by reductive work up with triphenylphospine afforded the target molecule **82** in 89% yield.

Herber and Breit synthesized Theodorakis’ C3-C11 fragment **82** of borrelidin with a 41% overall yield over eight steps, starting from the known precursor **122**.

### Synthetic studies of borrelidin

#### Iqbal’s strategy for constructing the C3–C17 fragment of borrelidin using cross-metathesis

**Scheme 20:**
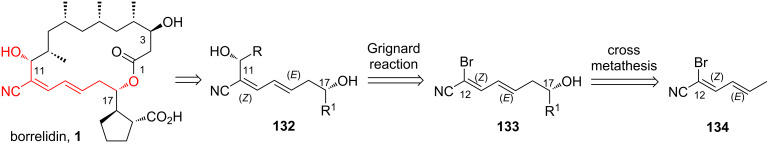
Retrosynthesis of the C3–C17 fragment of borrelidin by Iqbal and co-workers [35].

In 2008, Iqbal and co-workers conducted a synthetic study in which they successfully synthesized the C3–C17 fragment of borrelidin using a cross-metathesis reaction [35]. In the retrosynthesis, compound **132**, representing the synthetic target, was realized through an addition reaction of a Grignard reagent derived from **133** to the aldehyde counterpart (Scheme 20). Compound **133** was prepared via a cross-metathesis reaction of 2-bromohexa-2,4-dienenitrile **134** and a selection of alkenes.

**Scheme 21:**
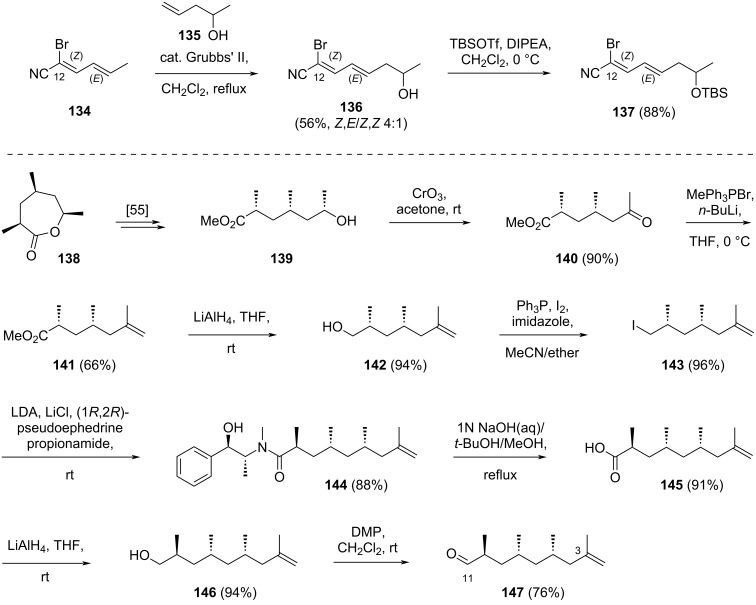
Synthesis of key intermediates **137** and **147** for the synthesis of the C3–C17 fragment of borrelidin.

Iqbal noted from the literature that cyano alkenes, such as acrylonitrile, predominantly yielded the *Z*-product during the cross-metathesis reactions. Therefore, the synthetic study was initiated by performing cross-metathesis reactions between both (*E*,*E*)- and (*Z*,*E*)-**134** with various alkenes to investigate the *Z*- or *E*-selectivity of the reaction. Fortunately, Iqbal observed that all cross-metathesis reactions in the study exhibited high *E*-selectivity. As a result, the reaction of (*Z*,*E*)-**134** with olefin **135** provided the desired product **136** in 56% yield, with a (*Z*,*E*)/(*Z*,*Z*) ratio of 4:1 (Scheme 21). Compound **136** was subsequently protected as a TBS ether, **137** [35].

The aldehyde counterpart **147** for the late stage coupling with **137** was prepared from the known lactone **138** (Scheme 21). Following a literature procedure, compound **139** was obtained from lactone **138** and subsequently treated with CrO_3_ to yield ketone **140** in 90% yield [55]. The ketone moiety of this compound was then reacted with a Wittig reagent derived from methyltriphenylphosphonium bromide and *n*-BuLi as base to provide olefin **141** in 66% yield. The ester group in this compound was reduced with LiAlH_4_ to afford primary alcohol **142** in 94% yield. The alcohol functionality was then converted to its corresponding iodide **143** (96%) upon treatment with Ph_3_P/I_2_/imidazole reagents and reacted with lithiated pseudoephedrine propionamide. The resulting product **144** was obtained in 88% yield. Basic hydrolysis of this compound successfully removed the chiral auxiliary, yielding acid **145** in 91% yield. Sequential reduction of this carboxylic acid with LiAlH_4_, followed by oxidation of the resulting primary alcohol with Dess–Martin periodinane, gave the anticipated aldehyde **147** in 76% yield over two steps.

**Scheme 22:**
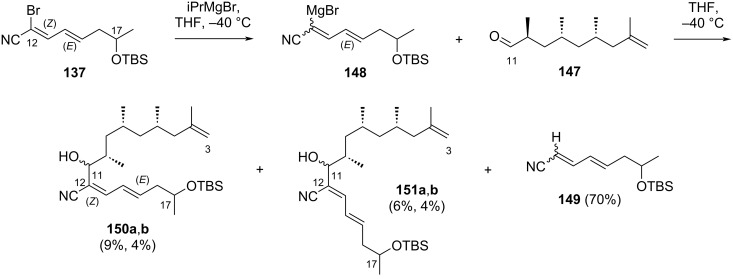
Synthesis of the C3–C17 fragment **150a**,**b** of borrelidin.

In the final stage, compound **137** was converted to organomagnesium intermediate **148** upon treatment with isopropylmagnesium bromide in THF and then reacted with aldehyde **147** (Scheme 22). After careful chromatographic purification, a pair of (*Z*,*E*)-**150a**,**b** (9%, 4%) corresponding to the C3–C17 fragment of borrelidin, as well as a pair of (*E*,*E*)-**151a**,**b** (6%, 4%), were isolated. Additionally, the debrominated product **149** was also isolated as the major product (70%).

In summary, Iqbal and co-workers synthesized the C1–C17 fragment of borrelidin using cross-metathesis as the main strategy. The desired compounds, **150a** and **150b**, were isolated in 2.8 and 1.2% yields, respectively, after 10 linear steps from compound **139**.

#### Iqbal’s strategy to construct the C11–C15 fragment of borrelidin and macrocyclization using a model compound based on metathesis reaction

**Scheme 23:**
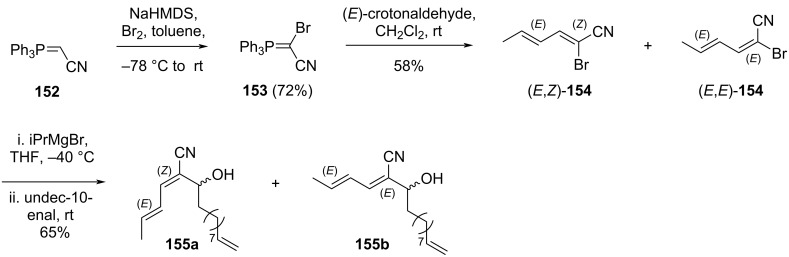
Synthesis of the C11–C15 fragment **155a** of borrelidin.

Two years earlier, in 2006, Iqbal and co-workers developed a strategy for synthesizing the C11–C15 fragment of borrelidin [34]. Additionally, they conducted a synthetic study to achieve the macrocyclization using a model system, employing a ring-closing metathesis reaction. As shown in Scheme 23, ylide **152**, derived from triphenylphosphine and chloroacetonitrile, was treated with bromine in the presence of sodium hexamethyldisilazide to afford compound **153** in 72% yield. Reaction of this intermediate with (*E*)-crotonaldehyde produced a mixture of (*E*,*Z*)- and (*E*,*E*)-isomers of **154** in a combined yield of 58%. Subsequent treatment of **154** with isopropylmagnesium bromide, followed by reaction with undecylenic aldehyde, provided compounds **155a** and **155b** (65% yield), representing the (*E*,*Z*)- and (*E*,*E*)-configurations, respectively.

**Scheme 24:**
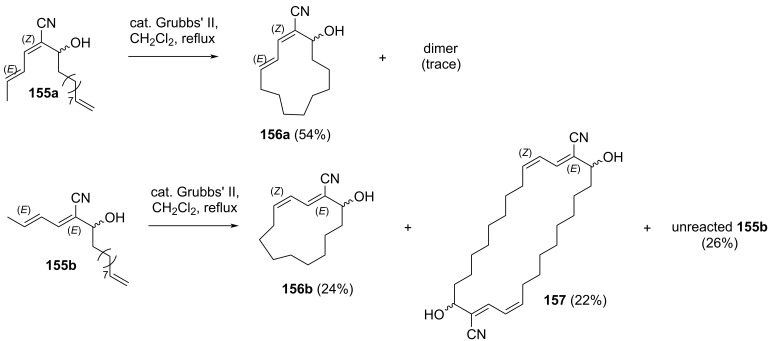
Macrocyclization of borrelidin model compounds **155a** and **155b** using ring-closing metathesis.

The macrocyclization study of borrelidin was subsequently carried out using model compounds **155a** and **155b** (Scheme 24). Heating compound **155a** in dichloromethane for 18 hours in the presence of 5 mol % Grubbs’ second generation catalyst yielded coupling product **156a** and unreacted starting material **155a**. Encouragingly, the addition of an extra 5 mol % of the catalyst followed by further heating for 28 hours successfully converted the remaining **155a** into **156a**, which was isolated in 54% yield. A trace amount of an unwanted dimer of **155a** was also detected. In contrast, different results were obtained when compound **155b** was used. Addition of 5 mol % catalyst produced the expected macrocyclic product and dimer **157** in yields of 24 and 22%, respectively, while a significant amount of unreacted **155b** (26%) remained under these conditions.

## Conclusion

In summary, this review has examined the various synthetic strategies employed in the construction of borrelidin fragments. By focusing on key intermediates and synthetic methods explored in recent literature, we have highlighted the feasibility and versatility of different approaches. These methods offer valuable insights into the efficient design and synthesis of borrelidin fragments, aiding the advancement of borrelidin-based drug development. Future research in this area should continue to explore novel synthetic strategies to optimize the synthesis and functionalization of borrelidin fragments, further supporting their potential applications in medicinal chemistry.

## Data Availability

Data sharing is not applicable as no new data was generated or analyzed in this study.
